# Association of the severity and progression rate of periodontitis with systemic medication intake

**DOI:** 10.3389/froh.2024.1447019

**Published:** 2024-08-02

**Authors:** Daniela Batista-Cárdenas, Agatha Araya-Castillo, María Paula Arias-Campos, Ana Paula Solís-Rivera, Jeniffer Jiménez-Matarrita, Lucía Piedra-Hernández, Luis Madriz-Montero, Karol Ramírez

**Affiliations:** ^1^ School of Statistics, University of Costa Rica, San José, Costa Rica; ^2^ Faculty of Dentistry, University of Costa Rica, San José, Costa Rica

**Keywords:** chronic diseases, diabetes mellitus, drug therapy, medication intake, oral-systemic disease, periodontitis

## Abstract

**Background/purpose:**

Information on the systemic medication profiles of patients with periodontitis is limited. Therefore, this retrospective cross-sectional study aimed to analyze the relationship between the severity and rate of progression of periodontitis and systemic medication intake using a database of patients who attended the Clinic of Periodontics of the Faculty of Dentistry of the University of Costa Rica.

**Methods:**

Electronic health records of patients diagnosed with periodontitis based on the Classification of Periodontal and Peri-Implant Diseases and Conditions (2017) were evaluated. Individuals were further categorized based on the severity (stage) and rate of progression (grade). Data extracted from the patient records included age, sex, and self-reported medication intake.

**Results:**

In total, 930 records were included. Most of the studied population was middle-aged (36–64 years old); 43.01% were male, and 56.99% were female. Four hundred and fifty-seven patients (49.14%) reported taking at least one systemic medication for a chronic condition. Regarding the periodontal treatment phase, 62.37% underwent steps 1–3, and 37.63% underwent step 4. The most common systemic medications taken were for cardiovascular diseases (42.28%), followed by medications for diabetes (14.46%) and neurologic disorders (14.46%). Most patients (59.35%) were diagnosed with Stage III periodontitis. Grade B (48.28%) was the most prevalent. Calcium channel blockers demonstrated a disease severity-dependent association with the periodontal stage (*p *= *0.021*). In addition, systemic medications for diabetes mellitus were associated with periodontal disease severity and rate of progression (all *Ps < 0.05*).

**Conclusions:**

This study provides indirect evidence of the association between systemic diseases and periodontitis. The positive association between medications used to treat diabetes and the severity and rate of progression of periodontitis may be due to the underlying disease rather than the medications *per se*.

## 1 Introduction

Periodontal disease is a chronic inflammatory condition initiated by microbial pathogens that destroy tooth-supporting components such as root cementum, periodontal ligament, and alveolar bone (1). In addition to the clinical attachment loss, other characteristics of periodontal disease include gingival bleeding, gingival margin recession, and periodontal pockets. In more Advanced-stage periodontitis may cause tooth mobility and loss if left untreated. Tooth loss may lead to masticatory dysfunction, speech alterations, and altered nutritional status, negatively impacting personal quality of life (2, 3).

Over the years, a bidirectional relationship has been established between diabetes and periodontitis (4). However, evidence for a causal relationship between periodontitis and other systemic diseases remains inconclusive. Nonetheless, consistent and robust epidemiological studies have suggested that periodontitis is a risk factor for adverse atherosclerotic cardiovascular disease events (5, 6). The association between periodontitis and many other diseases and conditions, including obesity, adverse pregnancy outcomes, respiratory disease, chronic kidney disease, rheumatoid arthritis, cognitive impairment, metabolic syndrome, and cancer, has been widely investigated (7, 8). The oral microbiome of susceptible hosts plausibly may shift to dysbiosis, initiating and promoting periodontal disease progression (9).

Research has focused on how specific systemic disease medications affect a healthy or inflamed periodontium (10). Generally, dampening the inflammatory pathways involved in the pathogenesis of periodontitis induces the oral side effects of drugs on the periodontium. Some medications can modify inflammatory and immunological responses of periodontal tissues to bacterial plaques (11). For example, gingival overgrowth has been reported in individuals consuming antiepileptics, immunosuppressants, and calcium channel blockers (12). Other drugs reported to affect the periodontium include corticosteroids, nonsteroidal anti-inflammatory drugs, and hormones (11). Systemic medications used to treat diabetes and hypertension are associated with lower salivary flow rates. The amount and composition of saliva are crucial first lines of defense against pathogens (13, 14). Although xerostomia does not cause periodontal disease, periodontal health may worsen in patients with dry mouth.

Limited information is available regarding the potential association between systemic medication intake and periodontitis. Earlier studies have not established an association between systemic medication intake and the severity and risk of periodontitis progression. Medication intake to treat noncommunicable diseases may reflect disease severity and indirectly portray the presence of systemic chronic inflammation. The current periodontal classification system can assess the association between staging, grading, and systemic medication intake. Furthermore, studies evaluating the periodontal state of Costa Ricans are scarce. Only one study describes the potential effect of self-reported medications on oral health in older Costa Ricans (15), and no studies have evaluated the relationship between the frequency of systemic medication intake and periodontitis in Costa Ricans. Therefore, we aimed to analyze the association between the frequency of systemic medication intake and the severity (stage) and risk of progression (grade) of periodontitis using a large database, such as the electronic health records (EHRs) of patients attending the Clinic of Periodontics at the University of Costa Rica from 2019 to 2023. Characterizing the systemic medication intake in patients with periodontitis may have important diagnostic and therapeutic implications. Our results could assist practitioners in providing more accurate and personalized treatment plans for patients with periodontitis. In other words, by providing more information on the risk factors related to the severity and progression of periodontitis, this targets bacterial biofilm disruptions, monitors patients’ overall health status, and identifies individuals with a higher risk of periodontal disease progression.

## 2 Materials and methods

### 2.1 Study design

This retrospective cross-sectional study was conducted according to the Strengthening the Reporting of Observational Studies in Epidemiology (STROBE) guidelines (16). This study was reviewed and approved by the Scientific Ethics Committee of the University of Costa Rica (CEC-283-2022) and conducted in accordance with the Helsinki Declaration of 1975, as revised in 2013.

We hypothesized that there is an association between systemic medication intake and the severity (stage) and risk of progression (grade) of periodontitis. To test this hypothesis, four student investigators (AAC, MPAC, APSR, and JJM) screened the EHRs of adult patients who attended the Clinic of Periodontics of the Faculty of Dentistry of the University of Costa Rica between January 2019 and December 2023.

### 2.2 Inclusion and exclusion criteria

Student investigators screened periodontal charts at the initial examination to select samples that met the inclusion criteria of the study protocol. The student investigators only included (1) patients with a diagnosis of periodontitis based on the classification scheme in the 2017 World Workshop on the Classification of Periodontal and Periimplant Diseases and Conditions (17), (2) patients aged 18 years and older, and (3) patients with completed health history questionnaire in the electronic health record.

Exclusion criteria included: (1) patients diagnosed with gingivitis or those with healthy periodontium.

### 2.3 Case definition of periodontitis

We included EHRs of patients with periodontal diagnoses based on the 2017 World Workshop on the Classification of Periodontal and Peri-Implant Diseases and Conditions. Eight periodontal specialists at the Clinic of Periodontics of the University of Costa Rica prepared the periodontal charts and performed the clinical examinations registered in the EHRs between January 2019 and December 2023.

The selected EHRs were further grouped into the following categories based on the severity and rate of periodontitis progression (18):

A. Severity of periodontitis:
  (i) Stage I (interdental clinical attachment loss of 1–2 mm or radiographic bone loss of <15%)
  (ii) Stage II (interdental clinical attachment loss of 3–4 mm or radiographic bone loss of 15%–33%)
  (iii) Stage III (interdental clinical attachment loss of ≥5 mm or radiographic bone loss extending to the middle third of the root and beyond. Showed ≤4 teeth loss due to periodontitis)
  (iv) Stage IV (interdental clinical attachment loss of ≥5 mm or radiographic bone loss extending to the middle third of the root and beyond. Showed ≥5 teeth loss due to periodontitis)
B. Rate of progression:
  (i) Grade A: slow rate (no bone loss or attachment loss over five years or percentage of bone loss/age is <0.25, affected are nonsmokers and those not diagnosed with diabetes).
  (ii) Grade B: Moderate rate [<2 mm bone loss or attachment loss over 5 years or percentage of bone loss/age is 0.25–1.0, and risk factors are those who smoke <10 cigarettes/day and have glycated hemoglobin (HbA1c) of <7.0%].
  (iii) Grade C: rapid rate (≥2 mm bone loss or attachment loss over 5 years or percentage of bone loss/age >1, smoking +10 cigarettes/day, and HbA1c ≥ 7.0% in patients with diabetes).

### 2.4 Data extraction

Data were extracted from the EHRs, including demographic characteristics (age and sex), smoking status, baseline or self-reported history of systemic medication intake at the initial visit, no systemic medication intake during the last 3 months as stated in the EHR, stage of periodontal treatment according to the European Federation of Periodontology (19, 20), and gingival bleeding index (GBI) (21) before Step 1 of periodontal therapy. Moreover, the plaque control record (PI) (22) was recorded before Step 1 and after Step 2 of periodontal treatment.

Baseline or first-visit self-reported intakes of systemic medications included those reported by Wang et al. (23). Generic names of medications or international nonproprietary names were used in the descriptive analysis to allow precise identification and communication. Antibiotics, corticosteroids, non-steroidal anti-inflammatory drugs, non-oral routes of administration, or an intake history of less than 3 months were excluded from the regression analysis. We excluded antibiotics, corticosteroids, and non-steroidal anti-inflammatory drugs since these medications are already known to affect clinical indicators of inflammation. Furthermore, only systemic medications administered orally were considered, since not all drugs offer non-oral routes of administration.

### 2.5 Statistical analysis

Data were tabulated, and statistical analysis was conducted in R (Version 4.0.3; R Core Team, 2020). Patient demographic and clinical characteristics are summarized as means and standard deviations or medians and interquartile ranges for continuous variables, as appropriate, and frequencies and percentages for categorical variables. To assess normality, a Quantile Plot was used to compare the theoretical quantiles of the data if they were perfectly distributed with normality and the quantiles of the measured values. The Shapiro–Wilk Test was used, where the null hypothesis was that the frequency distribution of the data was normally distributed. In this study, none of the variables met the assumption of normality.

The Mann–Whitney–Wilcoxon and chi-square tests were used to compare non-normally distributed and categorical outcomes, respectively. Associations between systemic medication intake variables and periodontal disease severity and progression rates were assessed using proportional odds regression models, with results presented as Odds Ratios (ORs) and corresponding 95% confidence intervals (CIs). Specifically, a multinomial ordinal logistic model was used to obtain ORs results. This method operates with the cumulative probabilities of Y when k = n and this seeks to identify the cumulative probability of being in different categorical combinations for the explained variable (Y), which is conditioned by the influence of X. Fisher's exact test was used because one of the expected frequencies was <5. Analysis of variance was used to detect differences in numerical variables. Proportional odds ratios were assessed using Brant's test. Multivariable regression models were used to explore the above outcomes, adjusting for the effects of age, sex, and smoking status at baseline. The *p*-values were corrected for multiple comparisons by using Holm's method.

## 3 Results

**Figure 1:**
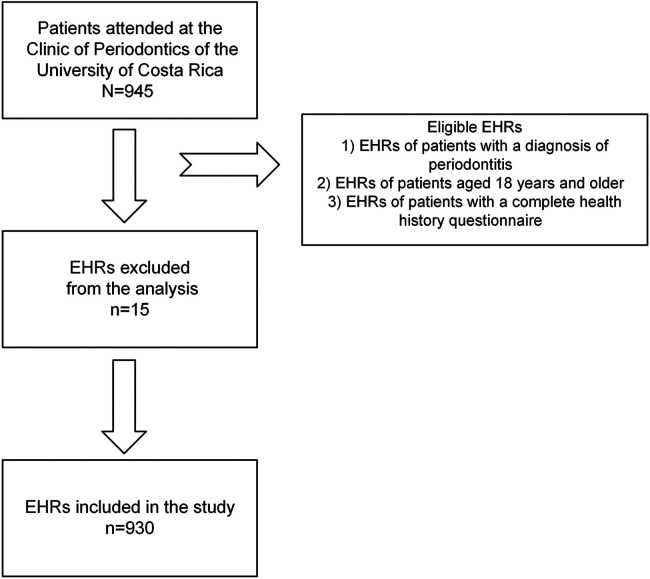
Flowchart showing the screening process of the included electronic health records (EHRs).

In total, 945 EHRs were reviewed. This study only included 930 EHRs of patients who received periodontal treatment at the Clinic of Periodontics of the Faculty of Dentistry at the University of Costa Rica. The 15 patient EHRs excluded from the analysis were those diagnosed with gingivitis or had healthy periodontium (Figure 1).

Table 1 summarizes the demographic characteristics and clinical indices of the study population. Of the 930 included records, 56.99% and 43.01% were from female and male patients, respectively *(p < 0.001).* The patients were categorized by age into young adults (aged 18–35 years; *n* = 8.82%), middle-aged adults (aged 36–64 years, *n* = 77.42%), and older adults (aged ≥65 years, *n* = 13.79%). For clarity, age categorization was based on the definition of “adulthood” by the American Psychological Association (24), with a slight modification. Young adults were categorized as persons aged 18–35 years, middle-aged adults from 36 to 64 years, and older people aged 65 years and above. We modified the age range of young adulthood because most are legally identified as adults at 18 years in Costa Rica.

**Table 1:** Demographic characteristics and clinical indices of the study population.

	No.	Percentage	*p-v*alue^a^
Overall demographic characteristics	930	100	
Age (years)			
18–35 (young)	82	8.82	***<0.001***
36–64 (middle-aged)	720	77.42	* *
≥65 (older adults)	128	13.79	* *
Sex			
Male	400	43.01	***<0.001***
Female	530	56.99	* *
Smoking			
Yes	228	24.52	***<0.001***
No	702	75.48	* *
Systemic medication			
No medication	473	50.86	*0.600*
Medication	457	49.14	
Treatment status			
Step 1,2,3	580	62.37	
SPC	350	37.63	
Periodontal indices^b^	Mean	SD	*p-v*alue^b^
Gingival bleeding	30.22	23.14	
Initial plaque index	65.60	18.06	***<0.001***
Final plaque index	30.82	18.46	

No, number; SPC, supported periodontal care; SD, standard deviation.

Bold *p*-values denote statistical significance at the *p* < 0.05 level.

^a^ Chi-square test.

^b^ Wilcoxon test.

Regarding cigarette smoking, 24.52% reported being active smokers, whereas 75.48% did not smoke *(p < 0.001)*. Half the participants reported not taking any systemic medication (50.86%), whereas the other half reported taking at least one medication to treat chronic diseases (49.14% *(p = 0.600).* In the periodontal treatment phase, 62.37% of the patients underwent steps 1–3, and 37.63% received supportive periodontal care. The initial GBI of the study population was 30.22 ± 23.14. The initial plaque index (PI) was 65.60 ± 18.06, and the final PI reported after the treatment conclusion was 30.82 ± 18.46 *(p < 0.001)*.

Supplementary Table S1 shows the medication types and consumption prevalence. The most common systemic medication reported was for cardiovascular disease (42.28%), followed by medications for neurological disorders (14.46%) and diabetes mellitus (14.46%). Some patients reported consuming more than one medication to treat chronic diseases.

Table 2 shows the demographic characteristics of the study population stratified by periodontal disease severity (stage). Most patients were diagnosed with stage III periodontitis (*n* = 552), followed by stage II periodontitis (*n* = 179), stage IV (*n* = 170), and stage I (*n* = 29). Univariable comparison tests indicated significant differences between the age groups *(p < 0.001)*. Middle-aged individuals were more likely to have stages II, III, and IV periodontitis *(p < 0.001)*. Periodontal disease severity between the sexes in the study population was not different *(p = 0.136)*. Differences in smoking status were also detected *(p = 0.021)*. People who smoked were diagnosed with stages III and IV periodontitis. In addition, the GBI differed between the stages, indicating that patients with stage IV periodontitis had the highest GBI index *(p = 0.022)*. Moreover, patients with stage IV periodontitis had a significantly higher initial PI before and after periodontal treatment *(p < 0.001, p = 0.011,* respectively*)*.

**Table 2:** Demographic characteristics of the study population by stage.

Demographic characteristics	Stage I No. (%) *n* = 29	Stage II No. (%) *n* = 179	Stage III No. (%) *n* = 552	Stage IV No. (%) *n* = 170	*p*-value^a^
Age (years)					
18–35 (young)	15 (51.72)	31 (17.32)	34 (6.16)	2 (1.18)	***<0.001***
36–64 (middle-aged)	14 (48.28)	133 (74.30)	439 (79.53)	134 (78.82)	* *
≥65 (older adults)	0 (0.00)	15 (8.38)	79 (14.31)	34 (20.00)	* *
Sex					
Male	18 (62.07)	76 (42.46)	228 (41.30)	78 (45.88)	*0.136*
Female	11 (37.93)	103 (57.54)	324 (58.70)	92 (54.12)	* *
Smoking					
Yes	3 (10.34)	39 (21.79)	131 (23.73)	55 (32.35)	***0.021***
No	26 (89.66)	140 (78.21)	421 (76.27)	115 (67.65)	* *
Periodontal indices	Stage I Mean (SD)	Stage II Mean (SD)	Stage III Mean (SD)	Stage IV Mean (SD)	*p*-value^b^
Gingival bleeding	26.65 (19.42)	28.72 (23.80)	29.40 (22.07)	35.07 (25.78)	***0.022***
Initial plaque index	58.51 (17.55)	64.15 (18.61)	64.99 (17.97)	70.33 (17.03)	***<0.001***
Final plaque index	27.26 (12.85)	28.09 (12.86)	30.82 (19.71)	34.32 (19.58)	***0.011***

No., number; %, percentage; n, sample size; SD, standard deviation.

Bold *p*-values denote statistical significance at the *p* < 0.05 level.

^a^ Chi-squared test.

^b^ Mann–Whitney–Wilcoxon test.

The demographic characteristics of the included population stratified by periodontal disease progression rates are shown in Table 3. Most included patients were diagnosed with grade B periodontitis (*n* = 449). Univariable comparison tests indicated significant differences in age categories at baseline between grades A, B, and C *(p < 0.001)*. Most individuals in the grades B and C periodontitis groups were middle-aged *(p < 0.001).* No significant difference was found between sex and periodontal disease progression *rate (p = 0.295)*. Regarding periodontal indices, the mean GBI and initial PI were higher in patients diagnosed with grade C than in those with grades A and B *(p < 0.001 and p = 0.002,* respectively*)*. Furthermore, the GBI decreased significantly after periodontal treatment at all the progression rates *(p < 0.001)*.

**Table 3:** Demographic characteristics and periodontal indices of the study population by disease progression rate.

Demographic characteristics	Grade A No. (%) *n* = 62	Grade B No. (%) *n* = 449	Grade C No. (%) *n* = 419	*p*-value^a^
Age (years)				
18–35 (young)	15 (24.19)	40 (8.91)	27 (6.44)	***<0.001***
36–64 (middle-aged)	44 (70.97)	331 (73.72)	345 (82.34)	* *
≥65 (older adults)	3 (4.84)	78 (17.37)	47 (11.22)	* *
Sex				
Male	30 (48.39)	182 (40.53)	188 (44.87)	*0.295*
Female	32 (51.61)	267 (59.47)	231 (55.13)	* *
Smoking				
Yes	9 (14.52)	93 (20.71)	126 (30.07)	***<0.001***
No	53 (85.48)	356 (79.29)	293 (69.93)	* *
Periodontal indices	Grade A Mean (SD)	Grade B Mean (SD)	Grade C Mean (SD)	*p*-value^b^
Gingival bleeding	23.55 (21.57)	27.92 (22.36)	33.68 (23.70)	***<0.001***
Initial plaque index	59.59 (17.60)	64.72 (18.28)	67.44 (17.67)	***0.002***
Final plaque index	27.98 (14.71)	28.86 (18.88)	33.34 (18.24)	***<0.001***

No., number; %, percentage; n, sample size; SD, standard deviation.

Bold *p*-values denote statistical significance at the *p* < 0.05 level.

^a^ Chi-squared test

^b^ Mann–Whitney–Wilcoxon test.

The association between systemic medication intake and the severity of periodontal disease (stage) is presented in Table 4. Multivariable proportional odds regression adjusted for age, sex, and smoking showed that calcium channel blocker usage was significantly associated with periodontal disease severity *(p = 0.021)*. Patients diagnosed with stages III and IV periodontitis reported consuming more systemic medications than those diagnosed with periodontitis stages I and II. The same analysis demonstrated that the intake of systemic medications for diabetes mellitus was significantly associated with the severity of periodontal disease *(p = 0.001)*. No association was found between the periodontitis stage and medication intake. Most ORs suggested a decreased risk or inverse correlation between periodontal stage and medication intake.

**Table 4:** Association between systemic medications and periodontitis stage.

Systemic medication intake	Overall	Stage I No. (%) *n* = 29	Stage II No. (%) *n* = 179	Stage III No. (%) *n* = 552	Stage IV No. (%) *n* = 170	Odds ratio (95% CI)	*p*-value^a^
No medication	473	17 (58.62)	103 (57.54)	268 (48.55)	85 (50.00)	1.24 (0.96, 1.59)	*0.162*
Medications for cardiovascular diseases	457	10 (34.48)	44 (24.58)	185 (33.51)	65 (38.24)	1.39 (1.06, 1.81)	***0.043***
ACE inhibitors	225	8 (27.59)	32 (17.88)	137 (24.82)	48 (28.24)	0.74 (0.55, 1.00)	*0.113*
Calcium channel blockers	66	0 (0.00)	9 (5.03)	36 (6.52)	21 (12.35)	0.47 (0.28, 0.76)	***0.021***
Diuretics	71	1 (3.45)	9 (5.03)	50 (9.06)	11 (6.47)	0.83 (0.52, 1.34)	*0.281*
Anticoagulants	0	0 (0.00)	0 (0.00)	0 (0.00)	0 (0.00)	0.00 (0.00,0.00)	*NA*
Blood-lipid lowering medications	98	1 (3.45)	18 (10.06)	58 (10.51)	21 (12.35)	0.80 (0.53, 1.20)	*0.594*
Alpha 2 antagonists	39	0 (0.00)	8 (4.47)	20 (3.62)	11 (6.47)	0.66 (0.35, 1.25)	*0.317*
Platelet antiaggregant	48	1 (3.45)	6 (3.35)	28 (5.07)	13 (7.65)	0.58 (0.33, 1.03)	*0.331*
Medications for neurologic disorders	104	0 (0.00)	22 (12.29)	63 (11.41)	19 (11.18)	1.06 (0.71, 1.59)	*0.224*
Antidepressants	79	0 (0.00)	21 (11.73)	42 (7.61)	16 (9.41)	1.05 (0.67, 1.65)	*0.112*
Anticonvulsants	17	0 (0.00)	3 (1.68)	10 (1.81)	4 (2.35)	0.72 (0.28, 1.85)	*0.934*
Antipsychotic drugs	27	0 (0.00)	9 (5.03)	15 (2.72)	3 (1.76)	1.67 (0.80, 3.50)	*0.274*
Medications for diabetes mellitus	104	3 (10.34)	8 (4.47)	64 (11.59)	29 (17.06)	2.05 (1.37, 3.07)	***0.001***
Insulin	19	1 (3.45)	0 (0.00)	10 (1.81)	8 (4.70)	0.28 (0.12, 0.69)	***0.010***
Oral hypoglycemic agents	98	3 (10.34)	8 (4.47)	60 (10.87)	27 (15.88)	0.50 (0.33, 0.76)	***0.004***
Medications for gastric pathologies/disorders	91	1 (3.45)	13 (7.26)	63 (11.41)	14 (8.24)	1.15 (0.75, 1.76)	*0.243*
Antiacids	86	1 (3.45)	13 (7.26)	58 (10.51)	14 (8.24)	0.86 (0.56, 1.33)	*0.446*
Proton pump inhibitors	5	0 (0.00)	0 (0.00)	5 (0.90)	0 (0.00)	0.88 (0.16, 4.96)	*0.542*
Medication for respiratory conditions/asthma	53	3 (10.34)	13 (7.26)	31 (5.62)	6 (3.53)	0.61 (0.36, 1.04)	*0.257*
Bronchodilators	53	3 (10.34)	13 (7.26)	31 (5.62)	6 (3.53)	0.61 (0.36, 1.04)	*0.257*
Thyroid disease	63	0 (0.00)	9 (5.03)	45 (8.15)	9 (5.29)	0.86 (0.52, 1.42)	*0.202*

No., number; %, percentage; n, sample size; CI, confidence interval.

Bold *p*-values denote statistical significance at the *p* < 0.05 level.

^a^ Fisher's exact test.

The associations between systemic medication intake and periodontal disease progression (grade) are shown in Table 5. Multivariable proportional odds regression, adjusted for age, sex, and smoking, revealed that the intake of systemic medications for diabetes mellitus *(p = 0.009)* was significantly associated with the periodontitis progression rate. Individuals who used insulin and oral hypoglycemic agents were more likely to have higher-grade periodontitis (*p = 0.006* and *p *= *0.030*, respectively). Most ORs suggested a decreased risk/inverse correlation between the periodontal grade and medication intake. The CIs of the antiacids, were affected due to the small sample size of patients diagnosed with Grade A. With a greater sample size the confidence interval would be narrower. If we had greater variability, the confidence interval would be wider. This is why the singificant *p* value is contradicting the CIs.

**Table 5:** Association between systemic medications and disease progression rate (grade).

Systemic medication intake	Overall	Grade A No. (%) *n* = 62	Grade B No. (%) *n* = 449	Grade C No. (%) *n* = 419	Odd ratio (95% CI)	*p*-value^a^
No medication	473	33 (53.22)	222 (49.44)	218 (52.03)	1.06 (0.83, 1.36)	*0.696*
Medications for cardiovascular diseases	457	19 (30.64)	151 (33.63)	134 (31.98)	0.92 (0.71, 1.20)	*0.825*
ACE inhibitors	225	15 (24.19)	112 (24.94)	98 (23.39)	1.07 (0.80, 1.43)	*0.877*
Calcium block channels	66	3 (4.84)	30 (6.68)	33 (7.88)	0.79 (0.49, 1.29)	*0.678*
Diuretics	71	3 (4.84)	41 (9.13)	27 (6.44)	1.23 (0.77, 1.96)	*0.257*
Anticoagulants	0	0 (0.00)	0 (0.00)	0 (0.00)	0 (0.00, 0.00)	*NA*
Blood-lipid lowering medications	98	10 (16.13)	42 (9.35)	46 (10.98)	1.01 (0.67, 1.51)	*0.226*
Alpha 2 antagonists	39	1 (1.61)	18 (4.01)	20 (4.77)	0.73 (0.39, 1.37)	*0.613*
Platelet antiaggregant	48	1 (1.61)	24 (5.34)	23 (5.49)	0.82 (0.46, 1.44)	*0.530*
Medications for neurologic disorders	104	6 (9.68)	53 (11.80)	45 (10.74)	0.92 (0.62, 1.36)	*0.879*
Antidepressants	79	6 (9.68)	41 (9.13)	32 (7.64)	1.22 (0.78, 1.90)	*0.671*
Anticonvulsants	17	1 (1.61)	7 (1.56)	9 (2.15)	0.74 (0.29, 1.89)	*0.855*
Antipsychotic drugs	27	2 (3.22)	15 (3.34)	10 (2.39)	1.35 (0.64, 2.85)	*0.665*
Medications for diabetes mellitus	104	3 (4.34)	40 (8.91)	61 (14.56)	1.82 (1.21, 2.73)	***0.009***
Insulin	19	1 (1.61)	3 (0.67)	15 (3.58)	0.22 (0.07, 0.67)	***0.006***
Oral hypoglycemic agents	98	3 (4.84)	39 (8.68)	56 (13.36)	0.57 (0.37, 0.86)	***0.030***
Medications for gastric pathologies/disorders	**91**	**1 (1.61)**	**54 (12.03)**	**36 (8.59)**	**0.89 (0.59, 1.35)**	***0.013***
Antiacids	86	1 (1.61)	52 (11.58)	33 (7.88)	1.13 (0.74, 1.74)	***0.014***
Proton pump inhibitors	5	0 (0.00)	2 (0.44)	3 (0.72)	0.51 (0.09, 3.07)	*0.771*
Medication for respiratory conditions/asthma	53	6 (9.68)	25 (5.57)	22 (5.25)	0.76 (0.44, 1.30)	*0.340*
Bronchodilators	53	6 (9.68)	25 (5.57)	22 (5.25)	0.76 (0.44, 1.30)	*0.340*
Thyroid disease	63	4 (6.45)	31 (6.90)	28 (6.68)	1.02 (0.62, 1.67)	*1.000*

No., number; %, percentage; n, sample size; CI, confidence interval.

Bold *p*-values denote statistical significance at the *p* < 0.05 level.

^a^ Fisher's exact test.

## 4 Discussion

To our knowledge, this is the first study to evaluate the potential association between systemic medication intake and periodontal disease severity and progression rate based on the 2017 World Workshop on the Classification of Periodontal and Peri-Implant Diseases and Conditions. This retrospective investigation included 930 EHRs of patients with periodontitis who reported their systemic medication intake. The results of the present study indicate (i) a disease severity-dependent association between calcium channel blockers and periodontal disease and no effect relating grade and (ii) an association between systemic medications for diabetes mellitus and periodontal disease severity and progression rate.

The results of the present study were in accordance with the 2017 Classification of Periodontal and Peri-Implant Diseases and Conditions, in which periodontitis cases were characterized using a two-vector system. The current classification of periodontal disease is reliable for describing complex factors and has lower susceptibility than other indices (25). Grade assessment involves identifying common risk factors for periodontitis. Describing the progression rate of periodontitis is crucial in epidemiological studies. The grade assessment describes basic demographic variables, current smoking status, number of cigarettes smoked per day, history of diabetes diagnosis, and metabolic control (25, 26). Furthermore, the current classification offers standardized case definitions for population-based surveillance of periodontitis.

Regarding sociodemographic data, the age group with the highest prevalence, severity, and rate of periodontitis progression in our analysis was middle-aged adults. Other clinical studies have demonstrated an increase in the prevalence and severity of periodontal disease with advancing age. This has been observed in adults aged 30–40 years, with increased exacerbations after 50 years (27–29). Globally, the incidence of severe periodontitis peaks around 38 years (30). Middle-aged and older individuals are more likely to develop periodontitis than young individuals because of multiple exposure factors, such as smoking, alcohol consumption, brushing frequency, and dental cleaning (31).

Most periodontal patients who attended our clinic were women. The scientific literature reports that women are more likely to visit their dentist and receive professional dental care than men (32, 33). Additionally, the periodontium undergoes an exaggerated inflammatory response to plaque, modified by female hormonal fluctuations during puberty and pregnancy, as oral contraceptive side effects and at the postmenopausal stage (34). Periodontitis has a higher prevalence and greater severity in men than in women (35, 36). Two large cross-sectional epidemiological investigations provided evidence of sexual dimorphism in destructive periodontal diseases (37, 38). Moreover, poorer oral hygiene behaviors have been reported in males compared to females (39). However, no clear relationship between sex and periodontitis has been identified.

Smoking is recognized as a risk factor for the onset, severity, and progression of periodontal disease (40–42). Current evidence indicates that smoking markedly influences multiple immunoinflammatory responses that contribute to dysbiosis in susceptible hosts and likely influences the severity and rate of progression of periodontitis (18, 43, 44). In line with well-established evidence, smoking was associated with the diagnosis of periodontitis stages III and IV, together with grade C in the current study. Most of our study population were nonsmokers, probably because the General Law on Tobacco Control and its Harmful Effects on Health, No. 9028, regulates smoking in public areas in Costa Rica. Moreover, the prevalence and consumption of tobacco cigarettes have decreased over the last few years owing to massive antismoking campaigns in Costa Rica (45).

The most common type of medication consumed by the study population was drugs used to treat cardiovascular diseases. In Costa Rica, cardiovascular diseases are the leading cause of death among non-communicable diseases, and high blood pressure has been associated with a 29% mortality rate (46). In the present investigation, patients diagnosed with stage III and IV periodontitis had a more frequent intake of calcium channel blockers. The association between calcium channel blockers and periodontal disease severity may be due to the influence of drug-induced gingival overgrowth (47). The mechanism underlying gingival overgrowth may be the stimulation of fibroblast proliferation and collagen production (48). To clarify, the present study did not include patients with a diagnosis of periodontal health or gingival diseases and conditions. The latter includes drug-influenced gingival enlargement.

Two other studies (23, 49) have also reported an association between calcium channel blockers and periodontitis. Contrary to our results, these studies found a positive association with other medications to treat cardiovascular diseases and periodontitis, which can be explained by the differences between our study design and those of the research participants. For example, a retrospective case-control study of patients seen in the Graduate Periodontics Clinic, School of Dentistry, University of Michigan, reported that the frequency of intake of ACE inhibitors, and diuretics was significantly higher in patients with periodontal disease than in healthy individuals (23). Recently, another study evaluating individuals from the greater Stockholm area, Sweden, reported a possible relationship between taking systemic medications, such as anticoagulants, ACE inhibitors, statins, and periodontitis stages III and IV (9). A longitudinal, database case-control study concluded that patients with periodontitis purchased more than 19 different subgroups of medications compared with healthy periodontal individuals, including calcium channel blockers, agents acting on the renin-angiotensin system, and statins (49).

The second most common type of systemic medication reported in this study was neurological disorders and diabetes mellitus. However, no association was found between the use of medications to treat neurological disorders and periodontitis severity. One of our limitations was that there are more than 1,000 neurological disorders to consider, and we only collected data on three types of medications used to treat these conditions from the EHRs. Wang et al. found that patients with periodontitis were more likely to consume antidepressants, anticonvulsants, and antipsychotic drugs than periodontally healthy controls (23). These researchers found a severity-dependent association with anticonvulsants. Contrastingly, Frankenhaeuser et al. reported no association between periodontitis and drug use (49). Future studies on the link between neurological disorders and periodontitis are required, considering the high incidence and detrimental effects of these conditions on the general population. Untreated periodontitis may induce a sustained systemic inflammatory stimulus that constantly activates microglia, causing neuroinflammation (50).

The prevalence of diabetes mellitus in Costa Rica is 14.8%, which is comparable to that in developed countries (51). Costa Rica's Social Security Fund covers approximately 90% of its population. Most patients are treated at the primary care level, and access to antidiabetic medications is limited to sulfonylureas, metformin, and human insulin (51). Diabetes mellitus exacerbates the severity of periodontal disease (52–56). Additionally, individuals with mild/moderate and severe periodontitis have higher HbA1c plasma levels than individuals without periodontitis (57). We found that in agreement with our hypothesis, insulin and hypoglycemic agents demonstrated a disease severity-dependent association when comparing periodontitis staging. Underlying diabetes is more likely to be associated with periodontitis severity than the medications analyzed.

Concerning the progression rate, the present study found a positive association between the consumption of drugs to treat diabetes and a higher grade of periodontitis. Insulin and oral hypoglycemic agents were associated with Grade C periodontitis because the current classification of periodontal disease includes HbA1c levels and smoking status as modifying factors that should be considered when determining the grading process. Diabetes Mellitus is a major risk factor for periodontitis progression (58, 59). A recent consensus has designated diabetes and periodontitis as comorbidities that accelerate each other's development and progression (60). Extensive evidence has indicated that periodontitis affects blood sugar control in patients with type 2 diabetes and aggravates diabetes-related complications (61–63). Consequently, periodontal treatment alone beneficially affects HbA1c levels and reduces inflammation. In addition, the promotion of diabetes control interventions, such as individual lifestyle counseling, dietary changes, and oral health education, are recommended for patients undergoing periodontitis therapy (20).

The present study had several strengths. We evaluated a large sample of patients diagnosed with periodontal disease at the Clinic of Periodontics of the Faculty of Dentistry at the University of Costa Rica. Of these patients, 50.86% reported not taking any medications, and 49.14% reported taking at least one medication to treat a systemic disease. Both groups allowed us to conduct detailed analyses. Using the current classification of periodontal disease, we found a link between a few systemic medications and the severity and progression rate of periodontitis. Previous studies have used pocket depth as a determinant, which may not entirely reflect the actual severity of periodontal disease. Another novelty is that our study targeted middle-aged individuals (35–64 years old). In addition, we included confounding factors such as smoking, age, and sex. However, we did not have enough information in the EHRs on other risk factors, such as stress and family history, for all patients included in the study. Therefore, we did not control for these variables.

Another limitation of our study is the association between the intake of two or more systemic medications and periodontal state. Hence, it would be interesting to conduct a follow-up study to analyze these possible associations. Another limitation was that not all patients reported the dose and dosage information in their medication profiles at their initial visit. Thus, this information was missing, and we could not further analyze these variables. Substantial variability in the dose of medications probably exists between patients. However, we did not assess the self-reported duration, underuse, or overuse of medications. Pharmacodynamic drug-drug interactions and periodontal disease are areas that have been sparsely studied. In a susceptible host, drug-drug interactions may act as predisposing and precipitating factors that trigger the onset and progression of periodontitis.

## 5 Conclusions

Within the limitations of this retrospective cross-sectional study, we suggest a relationship between the systemic medications used to control diabetes and severity and progression grade of periodontitis. It seems probable that the underlying chronic disease, in this case, diabetes, is more likely to be associated with periodontitis staging and grading than the medications analyzed *per se*. We found increased odds for medications for diabetes mellitus to both stage and grade, but decreased odds for insulin and oral hypoglycemic agents. This may be explained by the fact that the overall group included both treatments, insulin and oral hypoglycemic agents, adding the probabilities of both medications. It's important to keep in mind that there were patients that self-reported taking both drugs to control diabetes mellitus.

The same occurrence can be seen with medications for cardiovascular diseases and stage. Increased odds for systemic medications for cardiovascular diseases was assessed for rate of progression, but decreased odds were found when the medicaments were analyzed individually, except for calcium channel blockers. The overall group included all medicaments to treat cardiovascular diseases, adding each drug's probability. Moreover, there were patients that reported taking more than one medication to treat cardiovascular disease.

Our findings highlight the inextricable connection between oral and systemic general health and provide further evidence for dental and medical professionals to consider the indirect implications of systemic medications in the management and needs of periodontal patients.

## Data Availability

The raw data supporting the conclusions of this article will be made available by the authors upon request from the corresponding author.
