# Supplementary material for: Association of the severity and progression rate of periodontitis with systemic medication intake
Source: Front Oral Health. 2024 Aug 2;5:1447019. doi: 10.3389/froh.2024.1447019 (PMC11328918; doi:10.3389/froh.2024.1447019)
Supplement: Supplementary file 1 [file Table1.docx]

**Supplementary Table 1. Medication types and prevalence of consumption**

|  | **No.** | **Percentage** |
| --- | --- | --- |
| **Overall**  **Medications** | 719* | 100 |
| Medications for cardiovascular diseases | 304 | 42.28 |
| Medications for neurologic disorders | 104 | 14.46 |
| Medications for diabetes mellitus | 104 | 14.46 |
| Medications for gastric pathologies/disorders | 91 | 12.66 |
| Medication for respiratory conditions/asthma | 53 | 7.37 |
| Medication for thyroid disease | 63 | 8.76 |
| **Overall** | 194 | 100 |
| **Two medications** | 137 | 70.61 |
| Medications for cardiovascular diseases and  neurologic disorders | 17 | 8.76 |
| Medications for cardiovascular diseases and  diabetes mellitus | 51 | 26.29 |
| Medications for cardiovascular diseases and  gastric pathologies | 18 | 9.28 |
| Medications for cardiovascular diseases and  respiratory conditions asthma | 8 | 4.12 |
| Medications for cardiovascular diseases and  thyroid disease | 14 | 7.22 |
| Medications for neurologic disorders and  diabetes mellitus | 5 | 2.58 |
| Medications for neurologic disorders and  gastric pathologies/disorders | 8 | 4.12 |
| Medications for neurologic disorders and  respiratory conditions/ asthma | 7 | 3.61 |
| Medications for neurologic disorders and thyroid disease | 2 | 1.03 |
| Medications for diabetes mellitus  and gastric pathologies/disorders | 0 | 0.00 |
| Medications for diabetes mellitus  and respiratory conditions/asthma | 0 | 0.00 |
| Medications for diabetes mellitus  and thyroid disease | 4 | 2.06 |
| Medications for gastric pathologies and  respiratory conditions/asthma | 1 | 0.52 |
| Medications for gastric pathologies/disorders and thyroid disease | 1 | 0.52 |
| Medications for respiratory conditions/asthma and thyroid disease | 1 | 0.52 |
| **Three medications** | 46 | 23.71 |
| **Four medications** | 11 | 5.67 |

No. Number; *Considering there are patients that consume more than one medication.
